# Wide-Awake Local Anesthesia No Tourniquet in Adolescent Hand Surgery: A Systematic Review

**DOI:** 10.1016/j.jhsg.2025.100820

**Published:** 2025-08-29

**Authors:** Hannah Polley, Benjamin Blackman, John Tristan Cassidy, Johan van der Stok

**Affiliations:** ∗ School of Medicine, University of Limerick, Limerick, Ireland; † Department of Trauma & Orthopaedic Surgery, University Hospital Limerick, Limerick, Ireland

**Keywords:** Adolescents, Hand surgery, Local anesthesia, No tourniquet, Outcomes

## Abstract

**Purpose:**

Wide-awake local anesthesia no tourniquet (WALANT) allows for intraoperative assessment of function while minimizing systemic anesthesia effects and is frequently used in adult hand surgery. However, the safety and feasibility of WALANT in adolescents remains uncertain because of potential challenges with adherence and cooperation. This review aimed to compile the evidence for using WALANT in adolescents.

**Methods:**

PubMed, Medline, Embase, and Scopus were searched from inception to December 2024. Comparative and cohort studies were included. Outcomes, including procedure time, length of hospital stay, and pain scores, were analyzed. Because of the heterogeneity of outcomes investigated, a narrative review was performed.

**Results:**

Four studies, three case-control studies (n = 287) and one case series (n = 6), involving 166 WALANT surgeries were included. The mean age across all patients, including WALANT and control, was 14, with a range of 7–20 years old. The most common WALANT procedures were tendon repair (22.3%, n = 37) and ganglion removal (22.3%, n = 37), followed by digital nerve repair (18.1%, n = 30). One conversion (0.6%) to a general anesthetic was reported.

**Conclusions:**

Current evidence on the utilization of WALANT in adolescents is limited; WALANT may reduce procedure time, length of hospital stay, and improve pain scores.

Type of study/level of evidence: Therapeutic IV.

Wide-awake local anesthesia no tourniquet (WALANT) technique is widely accepted and used in adult hand surgery. WALANT uses local anesthetic (LA) and hemostatic agents, most commonly 1% lidocaine and 1:100,000 epinephrine, to achieve pain relief and bleeding control intraoperatively, without the need for sedation or tourniquets.^1^ Epinephrine serves a dual purpose in establishing hemostasis by inducing vasoconstriction and promoting platelet aggregation.^2^ Additionally, epinephrine delays the absorption of lidocaine, thereby prolonging anesthesia.^3^ Bicarbonate can be added as a buffer in a 1:10 mL ratio with lidocaine-epinephrine, reducing the acidity and thereby decreasing the pain associated with injection.^1^ To ensure adequate anesthesia and vasoconstriction, the anesthetic solution should be given 20–30 minutes prior to commencement of the surgery.^4^

This technique was first proposed by Lalonde,^5^ a Canadian hand surgeon, to reduce surgery wait times. Wide-awake local anesthesia no tourniquet offers numerous benefits for both surgeons and patients, as well as benefiting the health care system economically. The main benefits of WALANT include enhanced patient safety, greater access to surgery, and the ability to perform intraoperative diagnoses or assessments, which contribute to improved postoperative outcomes.^1^^,^^5^ Moreover, WALANT can be performed in various settings, including surgical rooms, clinics, and minor procedure rooms, making it readily accessible for patients. Historically, WALANT was primarily used for soft tissue procedures such as carpal tunnel and trigger finger releases. Its application has since expanded to include osseous procedures, such as carpal and distal radial fractures.^6^^,^^7^

Despite WALANT’s integration into the field of adult hand surgery, there remains limited evidence of its use in adolescent patients. The primary challenge to integrating WALANT in adolescents is adherence and compliance during the procedure.^8^^,^^9^ A thorough preoperative examination is essential to gauge whether the patient is a suitable candidate for WALANT. Both patients and their parents should be comfortable and confident with the technique. If patients are anxious, restless, fidgety, or needle-phobic, WALANT is generally not advisable. Moreover, the patient must be mature enough to understand the surgical process and remain still during the anesthetic injection. Distraction techniques, such as pinching or pressing on the skin, may aid in patient comfort. Additionally, to enhance patient autonomy, adolescents should be offered options during the procedure, such as listening to the surgeon explain each step, watching a show or movie, or listening to music. A minimal patient age of 10 years has been suggested.^8^^,^^9^

This review aimed to analyze the current literature on the use of WALANT in adolescent hand surgery, highlighting its potential advantages and areas for further research.

## Materials and Methods

The development of this research adhered to the preferred reporting items for systematic reviews and meta-analyses guidelines for coordinating systematic reviews and meta-analyses.^10^

### Search criteria

Four online databases (Embase, Medline, PubMed, and Scopus) were searched from inception to December 31, 2024. The goal was to identify studies examining the use of WALANT for hand surgery in adolescent patients. Comprehensive search terms including “WALANT,” “local anesthetic,” “pediatrics,” and “adolescent” were used. Appendix S1 outlines the complete search strategy used for this study. Adolescents were defined as individuals aged 7–20 years.

The research question and study eligibility were determined a priori. Studies were included if they met the following criteria: (1) any study design with a minimum of five patients, (2) examined the use of WALANT, (3) human studies, (4) patients aged 7–20 years, and (5) clinical and/or functional outcomes were reported. Studies were excluded if they: (1) included fewer than five patients, (2) were non-human studies, (3) included patients aged 6 years or younger or 21 years and older, or (4) did not examine the use of WALANT.

### Screening

Title and abstract screening were conducted independently and blindly by two authors (HP and BB). Discrepancies between reviewers were resolved through consensus. Full-text screening was conducted independently and blindly by the same two authors. Any conflicts with full-text screening were resolved through consensus.

### Assessment of agreement

Inter-reviewer agreement was evaluated using the κ-statistic for screening. A priori classification was determined using the following criteria: (1) κ of 0.91–0.99 was considered to be almost perfect agreement; (2) κ of 0.71–0.90 was considered to be considerable agreement; (3) κ of 0.61–0.70 was considered to be high agreement; (4) κ of 0.41–0.60 was considered to be moderate agreement; (5) κ of 0.21–0.40 was considered to be fair agreement; and (6) a κ value of 0.20 or less was considered to be no agreement.^11^

### Quality assessment

The methodological quality of nonrandomized studies was evaluated using the methodological index for nonrandomized studies (MINORS) criteria.^12^ Using the items on the MINORS checklist, noncomparative studies can achieve a maximum score of 16, whereas comparative studies can achieve a maximum score of 24. Noncomparative studies were categorized a priori as follows: (1) 0–4 indicates very low quality evidence, (2) 5–7 indicates low quality, (3) 8–12 indicates fair quality, and (4) scores ≥13 indicate high quality. For comparative studies, categorization was determined a priori as follows: (1) 0–6 very low quality, (2) 7–10 low quality, (3) 11–15 fair quality, (4) 16–20 good quality, and (5) ≥20 high quality.^13^

### Data abstraction

Data were extracted using an electronic spreadsheet (Google Sheets) designed a priori. Data extracted included characteristics of the studies (eg, author(s), publication year, and study design), demographic data of participants (eg, number of participants, participant age, and participant sex), study details (eg, surgical procedure), and outcomes (eg, procedure time, length of hospital stay [LOS], length of surgery, pain, patient reported outcome measures and conversion to general anesthesia).

### Outcome reporting and statistics

The primary outcome of this systematic review was the feasibility of the WALANT technique in adolescents. Secondary outcomes included the type of surgical procedure, surgical details, pain scores, patient reported outcome measures, and clinical outcomes. Pain during the procedure and anesthetic injection were reported. The surgical time, the time spent in the surgical room, and the LOS were reported. The visual analog scale (VAS) and number pain reporting scale (NPRS) were used to report pain. The VAS during activity was also reported, with results before and after surgery. The quick disabilities of the arm, shoulder, and hand questionnaire results were reported.^14^ Finally, grip strength was recorded before surgery and at follow-up. We qualitatively assessed heterogeneity by comparing key study characteristics, including patient age, indication for surgery, anesthesia technique, outcome measures, and duration of follow-up. Because of the heterogeneity in study designs and outcomes reported, pooled estimates and meta-analyses were not conducted, and the results were presented in a narrative fashion.

## Results

### Literature search

The literature search yielded 596 studies, with 321 duplicates removed. Of the 275 unique articles, 264 were removed following screening. Systematic screening yielded four full-text studies that satisfied inclusion criteria (Fig). Inter-rater reliability analysis showed substantial agreement at the screening stage (κ = 0.62; 95% CI, 0.36–0.87) and almost perfect agreement at the full-text stage (κ = 1.0; 95% CI, 1.0–1.0).

**Figure 1:**
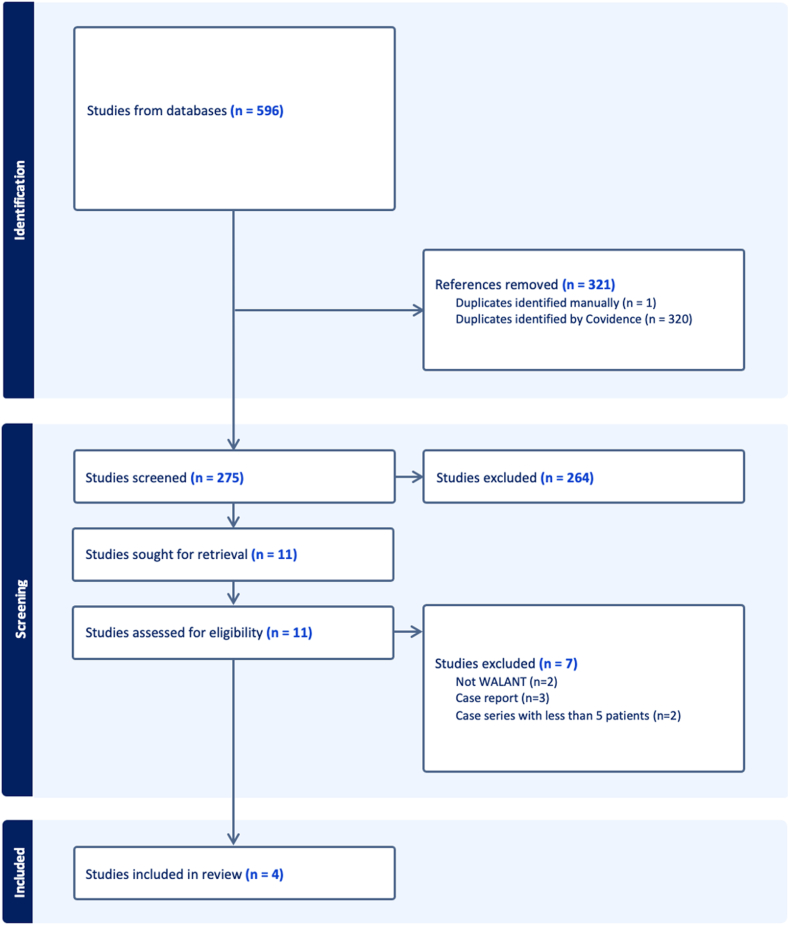
Preferred reporting items for systematic reviews and meta-analyses flow diagram representing a systematic review of WALANT in adolescent hand surgery.

### Study quality

The mean MINORS score was 20 (83%) for comparative and 12 (75%) for noncomparative studies. Individual scores are shown in the Table 1.

**Table 1:** Study Demographics

Authors	Mean MINORS Score	Study Design	Patients	Age (SD)	Male (%)
Tamburini et al^15^ (2025)	91.7%	Case-control	WALANT: 86 Control: 76	WALANT: 15 (2) Control: 14 (3)	WALANT: 36 (42%) Control: 39 (51%)
Long et al^16^ (2024)	91.7%	Case-control	WALANT: 28 Control: 28 (6 regional, 3 local + sedation, 19 GA)	WALANT: 15 (2) Control: 13 (2)	WALANT: 23 (82%) Control: 24 (86%)
Tuna and Ayhan^17^ (2022)	64.6%	Case-control	WALANT: 46 Control: 23 (GA)	WALANT: 15 (2) Control: 14 (2)	WALANT: 22 (48%) Control: 8 (35%)
Ozcelik and Sari^18^ (2021)	75%	Case Series	WALANT: 6	WALANT: 14 (12-19)	WALANT: 6 (100%)

### Patient characteristics

This review included four studies comprising 293 patients: three case-control studies (n = 287) and one case series (n = 6). Mean age was 14 (0.36), ranging from 7 to 20 years. There were 87 males (54%) and 79 females (48%) undergoing WALANT. Three studies included procedure location, with 124 (75%) of WALANT operations occurring in an outpatient or ambulatory setting.^15^, ^16^, ^17^ Patients were selected for the WALANT technique on a case-by-case basis. Suitability was assessed based on compliance and the anticipated duration of surgery.^15^, ^16^, ^17^, ^18^

### Surgery outcomes

The most common WALANT procedures were tendon repair (22.3%, n = 37) and ganglion removal (22.3%, n = 37), digital nerve repair (18.1%, n = 30), and open reduction internal fixation of phalangeal and metacarpal fractures (12.7%, n = 21). The most common procedures under general anesthetic (GA) were ganglion excision (26%, n = 33) and open reduction internal fixation (18.1%, n = 23). The surgical procedures are listed in the Table 2.

**Table 2:** Surgical Procedures Using the WALANT Technique and General Anesthesia

Surgical Procedures		Tamburini et al^15^ (2025)		Long et al^16^ (2024)		Tuna and Ayhan^17^ (2022)		Ozcelik and Sari^18^ (2021)		Total
		WALANT	Control	WALANT	Control	WALANT	Control	WALANT	Control	
Superficial soft tissue	Scar revision	-	-	2	1	-	-	-	-	3
	Hemangioma excision	-	-	-	-	1	1	-	-	2
	Foreign body excision	-	-	-	-	4	2	-	-	6
	Ganglion excision	32	30	-	-	5	3	-	-	70
	Tendon cyst excision	-	-	-	-	2	-	-	-	2
	Soft tissue tumor excision	20	13	-	-	-	-	-	-	33
	Trigger finger	-	-	-	-	6	-	-	-	6
	Irrigation/debridement	-	-	1	4	-	-	-	-	5
Deep soft tissue	Tendon repair	15	9	7	5	9	5	6	-	56
	Tenolysis	-	-	-	-	5	4	-	-	9
	Tendon transfer	-	-	-	-	2	1	-	-	3
	Pulley reconstruction	-	-	-	-	5	2	-	-	7
	Cubital tunnel release	-	-	-	-	1	-	-	-	1
	Digital nerve/artery repair	29	5	-	-	1	-	-	-	35
Bone	ORIF for metacarpal or phalangeal fractures	-	-	15	14	6	9	-	-	44
	CRPP for metacarpal or phalangeal fractures	6	10	-	-	-	-	-	-	16
	K-wire extraction	-	-	-	-	3	-	6	-	9
	Implant/hardware removal	4	10	3	2	-	-	-	-	19
	Finger amputation	-	-	-	2	-	-	-	-	2

CRPP, closed reduction percutaneous pinning; ORIF, open reduction internal fixation.

Tamburini et al^15^ (n = 162) found WALANT had significantly reduced mean total surgical time (34 ± 13 minutes WALANT vs 44 ± 25 minutes GA; *P =* .03) and length of surgery (22 ± 12 minutes WALANT vs 69 ± 22 minutes GA; *P =* .001). Few postoperative complications occurred; six patients (three WALANT and three GA) had recurrence of their original condition. Postanesthesia care unit costs were significantly lower for WALANT (569 ± 322 USD WALANT vs 1806 ± 714 USD GA; *P* = .001).

Long et al^16^ (n = 56) reported a reduced total room time (53 ± 23 minutes WALANT vs 64 ± 28 minutes GA; *P* = .05) and reduced LOS (222 ± 110 minutes WALANT vs 342 ± 204 minutes GA; *P* = .005). Surgical times were shorter with WALANT (43 ± 29 minutes) compared with GA (61 ± 38 minutes), although not statistically significant (*P* = .111). A list of surgical outcomes can be seen in the Table 3.

**Table 3:** Surgery Outcomes

Authors	Number of Cases in Hospital Operating Room	Surgical Time (Min)	Room Time (Min)	LOS (Min)	Estimated Postanesthesia Care Unit Expenses (USD)
Tamburini et al^15^ (2025)	WALANT: 14 (16%) Control: 32 (42%)	WALANT: 33.5 (12.8) Control: 44.0 (25.2) ***P* = .001**	WALANT: 22.0 (12.8) Control: 25.8 (22.3) ***P* = .03**	WALANT: 21.9 (11.8) Control: 69.0 (21.5) ***P* = .001**	WALANT: 569.0 (322.4) Control: 1805.9 (714.0) ***P* = .001**
Long et al^16^ (2024)	WALANT: 28 (100%) Control: 28 (100%)	WALANT: 43.9 (29.4) Control: 61.8 (38.9) ***P* = .111**	WALANT: 52.8 (23.2) Control: 63.7 (27.7) ***P* = .05**	WALANT: 222.1 (109.9) Control: 342.4 (203.8) ***P* = .005**	NR
Tuna and Ayhan^17^ (2022)	WALANT: 0 Control: 0	NR	NR	NR	NR
Ozcelik and Sari^18^ (2021)	NR	NR	NR	NR	NR

Bold text indicates statistical significance.

LOS, length of stay; NR, not reported; USD, United State dollars.

### Functional outcomes

Tamburini et al^15^ (n = 162) found that opioid prescriptions were significantly lower in WALANT (0.01%) compared with GA (0.07%). The NPRS scores were reduced at both 15 and 30 minutes after surgery (0.5 WALANT vs 1.2 GA at 15 minutes after surgery; *P =* .001; 0 WALANT vs 0.6 GA at 30 minutes after surgery; *P* = .001).

Tuna and Ayhan^17^ (n = 69) found that one patient (2.2%) required conversion to GA. Of the 46 WALANT patients, 16 were followed up. Three patients (18.7%) reported pain during LA injection (mean VAS 4.3/10); two patients (12.5%) reported intraoperative pain (mean VAS 3.5/10). Fourteen patients (87.5%) said they preferred WALANT if they required future hand surgery.

Ozcelik and Sari^18^ (n = 6) found improvement in VAS during activity (6.6 ± 1.3 before surgery to 0.2 ± 0.5 at follow-up; *P* = .018), the quick disabilities of the arm, shoulder, and hand scores (34.1 ± 18.4 before surgery to 3.9 ± 2.1 at follow-up; *P* < .001), and grip strength (22.7 ± 2.6 kg before surgery to 30.2 ± 4.2 kg at follow-up; *P* = .071).^18^ A summary of functional outcomes can be seen in the Table 4.

**Table 4:** Functional Outcomes

Authors	Opioid Pain Prescription	NPRS (15 Min Postop)	NPRS (30 Min Postop)	Conversion to GA	Pain (VAS) Injection	Pain (VAS) Procedure	Prefer WALANT	VAS (SD)	*Quick*DASH	Grip Strength (kg)
Tamburini et al^15^ (2025)	WALANT: 1 (0.01%) Control: 5 (0.06%)	WALANT: 0.5 Control: 1.2 ***P* = .001**	WALANT: 0.0 Control: 0.6 ***P* = .001**	0	NR	NR	NR	NR	NR	NR
Long et al^16^ (2024)	NR	NR	NR	0	NR	NR	NR	NR	NR	NR
Tuna and Ayhan^17^ (2022)	NR	NR	NR	1/46 (2.2%)	3/16 (4.3/10) Mean pain: 0.8/10	2/16 (3.5/10) Mean pain: 0.4/10	14/16 (87.5%)	NR	NR	NR
Ozcelik and Sari^18^ (2021)				0	NR	NR	NR	Rest: Preop: 1 (1.4) Postop: 0 (0) *P* = .076 Activity: Preop: 6.5 (1.3) Postop: 0.2 (0.5) ***P* = .018**	Preop: 34.1 (18.4) Postop: 3.9 (2.1) ***P* < .001**	Preop: 22.7 (2.6) Postop: 30.2 (4.2) *P* = .071

Bold text indicates statistical significance.

NPRS, number pain rating scale; NR, not reported; Postop, postoperative; Preop, preoperative; *Quick*DASH, quick disabilities of the arm, shoulder, and hand.

## Discussion

The primary finding of this review was that WALANT is a viable option for adolescents undergoing certain hand surgery procedures based on current evidence. Of the 166 WALANT patients included in this review, only one patient (0.6%) required conversion to GA.^17^ Two studies demonstrated a statistically significant reduction in total surgical room time.^15^^,^^16^ The largest case-control study included in this review found few postoperative complications, a significant reduction in opioid pain medication prescription, a significant reduction in NPRS scores both 15 and 30 minutes after surgery, and a significant reduction in postanesthesia care unit expenses for the WALANT group.^15^

The efficacy and safety of WALANT are well established in adult hand surgery.^19^, ^20^, ^21^, ^22^ A randomized study of 185 adult participants found that WALANT participants reported significantly less injection pain, reduced postoperative pain, lower use of analgesics after surgery, longer anesthetic duration, and overall greater patient satisfaction.^23^ Additional benefits to WALANT for adolescent patients include its possibility to use WALANT in patients with complex medical conditions that may preclude the use of GA, such as children with VACTERL who have congenital hand deformities and cardiac defects.^24^ Wide-awake local anesthesia no tourniquet does not require patients to alter or stop their medications before surgery, including anticoagulation or diabetic medications.^5^

Despite the advantages, implementing WALANT in a pediatric hospital remains challenging. Pediatric hospitals often strive to be pain-free environments.^16^ Recognizing the importance of minimizing pain in pediatric and adolescent surgery, several American hospitals, including Dartmouth Health Children’s, have implemented pain-free programs.^25^^,^^26^ These initiatives involve multidisciplinary teams—pediatricians, nurses, child life specialists, and anesthesiologists—who assess each child to create a personalized anesthetic plan. A key feature is flexibility, allowing adjustments if, for instance, a patient develops anxiety about the use of LA. These programs place an emphasis on parental involvement, ensuring parents believe empowered to advocate for their child.^26^ Tamburini et al,^15^ considered all patients for WALANT, but assessed eligibility on a case-by-case basis, considering age, procedure complexity, and patient and parent personality. The decision to proceed with WALANT was made collaboratively between the surgeon, patient, and parents. Additionally, child life specialists were present during the operation to provide comfort to the patients. Similar to pain-free programs, this approach emphasizes a case-by-case assessment, ensuring that patients and parents are actively involved in decision making and using an multidisciplinary team to provide individualized, patient-centered care.

Furthermore, injection of LA can be almost pain-free, using a specific technique that is easily teachable to medical students and residents, as has been shown by Farhangkhoee et al.^27^ After observing the correct injection technique, 76% of learners achieved a single pain event upon initial injection, on their first attempt, whereas 24% had patients experience pain twice. Notably, no learners caused more than two pain events.^27^ Additionally, the use of a 27- or 30-gauge needle and distraction techniques has been advised to reduce pain upon LA infection.^5^ All studies in this review referenced distraction techniques during injection of the LA, including deep breathing during injection and gentle proximal forearm pinching for sensory distraction.^15^, ^16^, ^17^, ^18^ Other techniques to minimize pain during injection include stabilizing the syringe with both hands, injecting tumescent LA in an antegrade fashion, and ensuring a 1 cm wheal of visible tumescence before advancing the needle distally.^17^ A recent study found that the use of noise-canceling headphones with music during WALANT hand surgery significantly decreased intraoperative patient anxiety.^28^ When implementing WALANT in adolescent patients, these aspects should be considered to minimize pain and enhance patient comfort.

Another important discussion when using LA in pediatric patients is the possible risk of toxicity because of reduced protein binding and underdeveloped hepatic clearance. Toxicity is influenced by both the absolute level of the LA and the rate of its rise in plasma concentration, with neonates being most vulnerable.^29^ Currently, epinephrine is the only approved adjunct for LA, enhancing block quality and duration while reducing side effects from high doses. For patients undergoing WALANT, the LA used in two of the included studies was 1% lidocaine with 1:100,000 epinephrine with a maximal dose of 7 mg/kg, another study used 0.5% bupivacaine premixed with epinephrine 1:200,000 with a maximal dose of 2.5 mg/kg, and the last study did not include their dosing.^15^, ^16^, ^17^, ^18^ The dosing regimens included in the studies in this review have been supported in the literature as safe.^1^^,^^5^

Currently, no standardized criteria exist to guide the use of WALANT in adolescent patients. Based on the findings of this review, we propose preliminary suitability criteria including patient age ≥10 years, ability to cooperate during consultation, anticipated surgical duration <60 minutes, and the use of intraoperative distraction methods (eg, headphones and tablets). These criteria may serve as a foundation for prospective studies aimed at developing guidelines for WALANT use in adolescents.

The strength of this systematic review was that it was a comprehensive analysis of the available studies on WALANT in adolescent hand surgery, albeit only including four studies. This review employed rigorous methodology and adhered to the preferred reporting items for systematic reviews and meta-analyses guidelines for systematic reviews. However, there are several limitations to this study. This review included only three comparative studies, all of which were retrospective.^15^, ^16^, ^17^ One of these studies had a significant loss to follow-up and lacked an adequate control group, as participants were asked which procedure they preferred. Furthermore, this study failed to report baseline characteristics to determine baseline equivalency of the comparative group.^17^ The fourth study included in this review only had six patients, limiting the power and generalizability of these findings.^18^

Furthermore, the heterogeneity in outcomes assessed in this review precluded our ability to perform a pooled analysis and calculate weighted means. Ultimately, these limitations highlight the scarcity of research on this topic. Future well-powered prospective studies with appropriate follow-up are needed to support the benefits of WALANT outlined in this review.

This review demonstrated that WALANT may improve hospital efficiency, reduce LOS and procedure time, and improve pain scores and functional outcomes in adolescents undergoing hand surgery.

## Conflicts of Interest

No benefits in any form have been received or will be received related directly to this article.
