# Supplementary material for: Wide-Awake Local Anesthesia No Tourniquet in Adolescent Hand Surgery: A Systematic Review
Source: J Hand Surg Glob Online. 2025 Aug 29;7(6):100820. doi: 10.1016/j.jhsg.2025.100820 (PMC12419090; doi:10.1016/j.jhsg.2025.100820)
Supplement: Supplementary Appendix [file mmc1.docx]

**Appendix 1.**

EMBASE: 16

('wide awake local anesthesia no tourniquet':ti,ab,kw OR walant:ti,ab,kw OR 'wide awake anesthesia':ti,ab,kw OR 'wide awake surgery':ti,ab,kw OR ('local anesthesia':ti,ab,kw AND 'no tourniquet':ti,ab,kw)) AND ('pediatrics'/exp OR 'pediatric':ti,ab,kw OR 'children':ti,ab,kw OR 'child':ti,ab,kw OR 'adolescents':ti,ab,kw OR 'adolescent':ti,ab,kw OR 'infant':ti,ab,kw OR 'infants':ti,ab,kw OR 'child'/exp)

MEDLINE: 18

1. ("Wide Awake Local Anesthesia No Tourniquet" OR WALANT).mp.
2. ("Wide Awake Anesthesia" OR "Wide Awake Surgery").mp.
3. ("Local Anesthesia" AND "No Tourniquet").mp.
4. 1 OR 2 OR 3
5. ("Pediatrics" OR "Pediatric" OR "Children" OR "Child" OR "Adolescents" OR "Adolescent" OR "Infant" OR "Infants").mp.
6. exp Pediatrics/
7. exp Child/
8. 5 OR 6 OR 7
9. 4 AND 8

PUBMED: 51

(("Wide Awake Local Anesthesia No Tourniquet"[Title/Abstract] OR WALANT[Title/Abstract] OR "Wide Awake Anesthesia"[Title/Abstract] OR "Wide Awake Surgery"[Title/Abstract] OR "Awake Surgery"[Title/Abstract] OR ("Local Anesthesia"[Title/Abstract] AND "No Tourniquet"[Title/Abstract]) OR ("Local Anesthesia"[Title/Abstract] AND Awake[Title/Abstract])) AND ("Pediatrics"[MeSH Terms] OR "Pediatric"[Title/Abstract] OR "Children"[Title/Abstract] OR "Child"[Title/Abstract] OR "Adolescents"[Title/Abstract] OR "Adolescent"[Title/Abstract] OR "Infant"[Title/Abstract] OR "Infants"[Title/Abstract] OR "Child"[MeSH Terms]))

SCOPUS: 154

(TITLE-ABS-KEY("Wide Awake Local Anesthesia No Tourniquet") OR TITLE-ABS-KEY(WALANT) OR TITLE-ABS-KEY("Wide Awake Anesthesia") OR TITLE-ABS-KEY("Wide Awake Surgery") OR TITLE-ABS-KEY("Awake Surgery") OR (TITLE-ABS-KEY("Local Anesthesia") AND TITLE-ABS-KEY("No Tourniquet")) OR (TITLE-ABS-KEY("Local Anesthesia") AND TITLE-ABS-KEY(Awake))) AND (TITLE-ABS-KEY(Pediatrics) OR TITLE-ABS-KEY(Pediatric) OR TITLE-ABS-KEY(Children) OR TITLE-ABS-KEY(Child) OR TITLE-ABS-KEY(Adolescents) OR TITLE-ABS-KEY(Adolescent) OR TITLE-ABS-KEY(Infant) OR TITLE-ABS-KEY(Infants))
